# 2,500-year Evolution of the Term Epidemic

**DOI:** 10.3201/eid1206.051263

**Published:** 2006-06

**Authors:** Paul M.V. Martin, Estelle Martin-Granel

**Affiliations:** * Institut Pasteur de Nouvelle Calédonie, Nouméa, New Caledonia;; † Collège Enseignement Secondaire Le Bosquet, Bagnols-sur-Cèze, France

**Keywords:** epidemic, history of the word, semantic evolution

## Abstract

The term epidemic (from the Greek *epi* [on] plus *demos* [people]), first used by Homer, took its medical meaning when Hippocrates used it as the title of one of his famous treatises. At that time, epidemic was the name given to a collection of clinical syndromes, such as coughs or diarrheas, occurring and propagating in a given period at a given location. Over centuries, the form and meaning of the term have changed. Successive epidemics of plague in the Middle Ages contributed to the definition of an epidemic as the propagation of a single, well-defined disease. The meaning of the term continued to evolve in the 19th-century era of microbiology. Its most recent semantic evolution dates from the last quarter of the 20th century, and this evolution is likely to continue in the future.

## Abstract 2

The meaning has been evolving since Hippocrates first used this word 25 centuries ago.

At the start of the 21st century, epidemics of infectious diseases continue to be a threat to humanity. Severe acute respiratory syndrome, avian influenza, and HIV/AIDS have, in recent years, supported the reality of this threat. Civil wars and natural catastrophes are sometimes followed by epidemics. Climate change, tourism, the concentration of populations in refugee camps, the emergence of new human pathogens, and ecologic changes, which often accompany economic development, contribute to the emergence of infectious diseases and epidemics (*1*). Epidemics, however, have occurred throughout human history and have influenced that history. The term epidemic is ≈2,500 years old, but where does it come from?

## Before Hippocrates

When works that put forward new ideas are translated, determining the original terminology (in Ancient Greek in this case) is not easy. In 430 BC, when Hippocrates was collecting the clinical observations he would publish in Epidemics, his treatise that forms the foundation of modern medicine, at least 3 terms were used in Ancient Greece to describe situations that resembled those described by Hippocrates: *nosos*, *phtoros*, and *loimos* (*2*).

Nosos, meaning disease, was used by Plato in the 4th century BC and clearly had the same meaning 2 centuries earlier in the works of Homer and Aeschylus. Nosos encompasses disease of the mind, body, and soul: physical, including epilepsy, and moral (i.e., psychological and psychiatric). Phtoros or *phthoros* means ruin, destruction, deterioration, damage, unhappiness, and loss, after war for example. The word was frequently used by Aeschylus and Aristophanes, was known in the 8th century BC, and was later used by Plato and Thucydides. Its meaning has remained general. Bailly translates loimos as plague or contagious scourge. Used by Esiodus in the 7th century BC and later by Sophocles and Herodotus, this term is ancient. Its translation as plague should be interpreted in the sense of a scourge rather than as the disease plague. In the Septuagint, a translation of the Old Testament into Greek by 70 Greek Jews from Alexandria, this word is used in the book of Kings to describe the 10 plagues of Egypt.

But the term epidemic already existed in 430 BC. The Greek word *epidemios* is constructed by combining the preposition *epi* (on) with the noun *demos* (people), but demos originally meant "the country" (inhabited by its people) before taking the connotation "the people" in classical Greek. Indeed, the word epidemios was used by Homer, 2 centuries before Hippocrates, in the Odyssey (canto I, verses 194 and 230), where it was used to mean "who is back home" and "who is in his country" in contrast to a voyager who is not: δὴ γάρ μιν ἔφανι ’ἐπιδήμιον έπιδήμιον εἶναι σὸν πατέρα_,_ "because someone said that your father was back (home)" (canto I, verse 194). In this context, epidemios means indigenous or endemic. In the Iliad, Homer confirmed this meaning (canto XXIV, verse 262), by using also *polemos epidemios* to mean civil war: ὃς πολέμου ἔραται ἐπιδημίου οκρυόευτος, "this one who liked passionately the frightening civil war" (canto IX, verse 64). Later, Plato and Xenophon (400 BC) used the word to describe a stay in a country or the arrival of a person: Παριος ὅυ έγὼ ᾐσθόμην έπιδημοῡντα, "a Parian who, I learned, was in town" (Plato, Apology, chapter I, paragraph 38). The verb *epidemeo* was used by Thucydides (460 BC–395 BC) to mean "to stay in one's own country," in contrast to *apodemeo*, "to be absent from one's country, to travel." For Plato, epidemeo meant "to return home after a voyage, to be in town." Later, the orators Demosthenes (384 BC–322 BC) and Eschines (390 BC–314 BC) used this word to refer to a stranger who came to a town with the intention of living there, and the verb epidemeo was used to mean "to reside." Typical of Greek semantics, epidemeo takes its meaning from the result of the action, rather than from the action itself. It relates to something that has already happened, with the implication that it had previously happened elsewhere. Authors before Hippocrates used epidemios for almost everything (persons, rain, rumors, war), except diseases. Hippocrates was the first to adapt this word as a medical term.

## Hippocrates and the Term Epidemic

Written in the 5th century BC, Hippocrates' Corpus Hippocraticum contains 7 books, titled Epidemics (*3*). Hippocrates used the adjective epidemios (on the people) to mean "which circulates or propagates in a country" (*4*). This adjective gave rise to the noun in Greek, *epidemia*.

We do not know why Hippocrates chose epidemios to title his books instead of nosos, a well-established term meaning disease. Examining the meaning of the term before, during, and after his time may help us understand his choice. Schematically speaking, epidemios (or epidemeo) was used successively to mean "being at homeland" (Homer), "arriving in a country" or "going back to homeland" (Plato), and later "stranger coming in a city" (Demosthenes). Sophocles (495 BC–406 BC) used the adjective in Oedipus Tyrannos to refer to something (a rumor, noise, fame, or reputation) spreading in a country: εῑμ’ Οἰδιπόδα ἐπὶ τ’αν ἐπίδαμον φ’ατιν, "I shall go (to make war) to Oedipus, against his fame which spread (in the country)" (verse 494). Oedipus Tyrannos was written at approximately the same time as Corpus Hippocraticum; consequently, we can infer that during Hippocrates' time, epidemios acquired a dynamic meaning, probably more adapted to describing a group of physical syndromes that circulate and propagate seasonally in a human population (i.e., on the people) than nosos, a term used to describe diseases at the individual level.

How epidemios, meaning "on the people," became adapted to mean "that which circulates or propagates in a country" is a crucial question. This evolution occurred during the second half of the 5th century (450 BC–400 BC), a period of intense activity in Greek literature, particularly with the prolificacy of Sophocles. But while nosos or loimo*s* were frequently used, epidemios was not. In the Perseus Digital Library (http://www.perseus.tufts.edu), a database that does not yet include Hippocrates' works, the adjective epidemios was used only 9 times, including 4 times in Homer, in the 489 major referenced Greek texts (≈4.8 millions words, 0.02 occurrences per 10,000 words). Its Doric variants *epidamos* and *epidemos* were used 3 times. In comparison, nosos (disease) was used 712 times in the Perseus database (1.47 occurrences per 10,000 words). The verb epidemeo was used 144 times, primarily during the 4th century and always meaning "to live in or return to one's own country." This lack of material makes accurately exploring the reasons for the semantic evolution across centuries difficult. In Oedipus Tyrannos, Sophocles qualified the sense of epidemios as it referred to reputation or fame; fame naturally spreads in a country. But Hippocrates described a series of syndromes: καὶ γαρ άλλως το νούσημα ἐπίδημον ἦν, "It is a fact that the disease was propagating in the country" (Epidemics, book I, chapter 3). Although Sophocles used epidemios once in that new sense, Hippocrates established a medical meaning for the term.

In Epidemics, books I and III constitute lists of diseases describing clinical cases. Hippocrates compared these cases and grouped them to generate series of similar cases. He adopted a classification approach, initially seeking clinical similarities between cases, thereby discovering, in addition to the notion of epidemic, the more fundamental concepts of symptom and syndrome. However, Hippocrates believed that prognosis was a major aspect of medicine. This belief led him to consider disease a dynamic process with its own progression, a temporal dimension, that represents a first nosologic evolution: syndromic groupings become diseases. Another of the books written by the physician from Kos—Airs, Waters, and Places—deals with the relationships between diseases and the environment, focusing particularly on the habitat of the patients and the season in which disease occurs. Hippocrates tried to determine the effect of environmental factors on what could be described as the distribution of diseases. He was, thus, more concerned about grouping together winter diseases or autumn diseases or diseases that occurred in a particular place or in persons whose way of life had changed than in identifying a large number of cases of the same disease in winter or autumn, at a particular place, or in association with a particular way of life. For Hippocrates, whose nosologic approach already contained a major element of preoccupation with the environment, the first meaning of epidemic was groups of cases resembling each other clinically and the second meaning was groups of different diseases occurring at the same place or in the same season and sometimes spreading "on the people." Thus, Hippocrates applied the word epidemios to groupings of syndromes or diseases, with reference to atmospheric characteristics, seasons or geography, and sometimes propagation of a given syndrome in the human population.

Semantic confusion caused the great Emile Littré, who translated Hippocrates' works into French in the first half of the 19th century, to make a nosologic error. Hippocrates described what is known today, since the work of Littré, as the Cough of Perinthus. This account can be found in Epidemics book VI. Hippocrates described coughs that started toward the winter solstice and were accompanied by many symptoms: sore throat, leg paralysis, peripneumonia, problems with night vision, voice problems, difficulty swallowing, difficulty breathing, and aches. When Littré published his translation and commentaries on Epidemics in 1846, he mistakenly considered the Cough of Perinthus to be a single disease (*5*). This error made retrospectively diagnosing the diseases of Perinthus difficult, if not impossible. Moreover, as Littré saw this collection of illnesses as a single disease, he essentially turned it into an epidemic, probably because he had the modern sense of the term in mind and thought that Hippocrates had observed and described an epidemic illness unknown to modern medicine (*5*).

According to Grmek, "Littré took chapter VI, 7.1 as a general description of an epidemic in the sense of this word in the medical language of the 19th century rather than in the sense intrinsic to the works of Hippocrates. In the Corpus Hippocraticum, the noun ‘epidemic' designates a collection of diseases observed at a given place, during a given period. A disease described as epidemic, such as epidemic cough, is a condition occurring from time to time in a given place, the appearance of which is closely linked to changes in season and climatic variations from year to year" (*5*).

Historians of medicine and philologists have over the years attributed the Cough of Perinthus to diphtheria, influenza, epidemic encephalitis, dengue fever, acute poliomyelitis, and many other diseases. However, a French physician named Chamseru, who practiced in the 18th century, almost a century before Littré, finally got to the bottom of what may be meant by the Cough of Perinthus, probably because the term epidemic had not yet taken on the meaning it had in Littré's time. According to Chamseru, the Cough of Perinthus could have encompassed several diseases, among them diphtheria, influenza, and whooping cough (*5*).

## Thucydides and the First Descriptions of Epidemics

Thucydides (460 BC–395 BC) interrupted his account of the Peloponnesian War to describe the famous Plague of Athens, which occurred at the start of the summer in 430 BC. This description was long considered among the first descriptions of an epidemic. Indeed, whereas Thucydides used nosos, the term plague, which is used by all the translators of his work, is used in the sense of the Latin term *pestis*, a term with no clear etymology (*4*), meaning contagious disease, epidemic, or scourge. The description of the Plague of Athens, like that of the Cough of Perinthus by Hippocrates, is an essential text in the philologic and semantic study of epidemics (*5*). We must therefore consider, as for Littré's translation, the meaning that translators have assigned to the original description by Thucydides. Thucydides never used the term epidemic that Hippocrates was in the process of establishing. Under the term nosos, Thucydides described a series of clinical signs, which originated in the south of Ethiopia and propagated throughout Egypt, Libya, and then Greece. Thucydides used the words nosos, *kakos* (evil), *ponos* (pain), phtoros (ruin, destruction), and loimos (scourge) to describe what his translators call plagues. In her translation of Thucydides' works (*6*), published in 1991, in the chapter titled Second Invasion of Attica: the Plague of Athens (the original Greek work had no title), Jacqueline de Romilly translated nosos as disease or epidemic. Similarly, she translated loimos, kakos, and phtoros as disease or epidemic and the list of clinical signs (the original Greek meant "following these things") as symptoms. de Romilly rendered the text more elegant and accessible to 20th-century readers by this translation but gave the words used by Thucydides their 20th-century meaning rather than the meanings they had in the 5th century BC. Herein lies the principal problem of translation.

But was the Plague of Athens a true epidemic, in the modern sense? The death rate for the disease was extremely high, reaching up to 25% in 1 group of soldiers, and Pericles died of it. Historians have tried to understand the origin of this plague, and various diseases have been suggested, e.g., typhus, measles, smallpox, bubonic plague, ergotism, or an unknown disease. Thucydides wrote that all preexisting diseases were transformed into a plague and that persons in good health were affected in the absence of a predisposing cause (*7*). The large number of symptoms and of possible and probable causes rules out the possibility of an epidemic in the modern sense of the term. Instead, the Plague of Athens seems to have been the appearance of a large number of diseases that affected the population at the same time. Plague therefore has the same meaning here as epidemic in the works of Hippocrates. These 2 terms have been used in association or confused throughout history. However, epidemic existed at this time, even if the notion of epidemic as we mean it in modern times had not yet emerged.

## Evolution of the Term Epidemic

After the nonmedical use of the term epidemic by Homer, Sophocles, Plato, and Xenophon, Hippocrates gave it its medical meaning. However, the term has since undergone a long evolution. The adjective epidemios gave rise to the Greek noun *epidemia*. The Greek term epidemia in turn gave rise to the Latin term *epidimia* or *epidemia*. The term *ypidime* in Medieval French has its origins in these Latin words and went on to become *épydime* in the 14th century, *epidimie* in the 17th century, and then *epidémie* in the 18th century. Not until 22 centuries after Hippocrates, in the second half of the 19th century, were the terms *épidémiologie* (1855), *épidémiologique* (1878), and *épidémiologiste* (1896) coined in French and notions attached to them developed. Of course, at approximately the same time, corresponding terms appeared in the English language. The term epidemic and the terms linked to it therefore required an extremely long time to be constructed. This evolution is representative of the evolution of science and medicine over the centuries and reflects the semantic evolution of the term.

## Semantic Evolution

In parallel with the evolution of the term epidemic itself, its meaning also changed over time. If we limit ourselves to the meaning that epidemic has acquired with respect to infectious diseases, we can identify 4 major steps in its semantic evolution in the medical sense. For Hippocrates, an epidemic meant a collection of syndromes occurring at a given place over a given period, e.g., winter coughs on the island of Kos or summer diarrheas on other islands. Much later, in the Middle Ages, the long and dramatic succession of waves of The Plague enabled physicians of the time to identify this disease with increasing precision and certainty; they began to recognize epidemics of the same, well-characterized disease. Then, with the historic contributions of Louis Pasteur and Robert Koch, epidemics of a characteristic disease could be attributed to the same microbe, which belonged to a given genus and species. The last stage in the semantic evolution of the term epidemic was the progressive acquisition of the notion that most epidemics were due to the expansion of a clone or clonal complex of bacteria or viruses known as the epidemic strain (*8*). More recently, microevolution of a clone of a bacterium (the epidemic strain) was shown to occur during an epidemic with person-to-person transmission (*9*). The Table summarizes these 4 major stages in the semantic evolution of the term epidemic.

**Table:** Semantic evolution of the term epidemic

Stage in evolution	Meaning	Use
Greek: *epi* (on) and *demos* (people) (6th century BC); *epidemios* used by Homer in the Odyssey	Who is in his country	Nonmedical use
Greek: Sophocles and Hippocrates (second half of the 5th century BC)	That which circulates and propagates in a country	First medical use
Greek: *epidemios* established by Hippocrates (430 BC) in the medical sense of a collection of syndromes	Sometimes spreading "on the people"	Epidemic of diarrhea
Medieval French: *ypidime* (1256 and later, *epidimie*)	Large number of cases of unique, well-characterized disease	Epidemic of cholera
19th century: *épidémie* (late 18th-century French) and *epidemic* (18th-century English)	Epidemics caused by a microbe belonging to a given genus and species	Epidemic of cholera due to *Vibrio cholerae*
End of 20th century	Clonal expansion of an epidemic strain, as defined with molecular markers	An epidemic due to *V. cholerae* El Tor, belonging to a defined ribotype or pulsotype

In the second half of the 20th century, epidemic was also applied to noninfectious diseases, as in cancer epidemic or epidemic of obesity. The extension of the meaning to noninfectious causes refers to a disease that affects a large number of people, with a recent and substantial increase in the number of cases. This semantic extension of epidemic also concerns nonmedical events; the term is used by journalists to qualify anything that adversely affects a large number of persons or objects and propagates like a disease, such as crack cocaine or computer viruses.

What can we gain from investigating the origin and meaning of the word epidemic or from studying its semantic evolution? Beyond simply satisfying our curiosity, the slow evolution of the form and meaning of the term suggests that we still have much to learn about the concept of epidemic.
